# Protocol for labeling and fixation of intact lysosomes with esterified amino acid analogs to assess lysosomal expansion in living eukaryotic cells

**DOI:** 10.1016/j.xpro.2021.100916

**Published:** 2021-10-27

**Authors:** Gianluca Scerra, Maria Gabriella Caporaso, Maurizio Renna, Massimo D’Agostino

**Affiliations:** 1 Department of Molecular Medicine and Medical Biotechnologies, University of Naples ‘‘Federico II’’, 80131 Naples, Italy

**Keywords:** Cell Biology, Microscopy, Molecular/Chemical Probes

## Abstract

The lysosomal compartment is a key hub for cell metabolism, and morphological alterations have been described in several pathological conditions. Here, we describe the use of amino acid analogs modified by the presence of a methyl ester group that accumulates within lysosomes. This generates an intraluminal osmotic effect able to trigger a rapid and selective expansion of the lysosomal compartment within 2 h of treatment. We also present protocols to preserve lysosomal morphology, which yields a more accurate size measurement.

For complete details on the use and execution of this protocol, please refer to Scerra et al. (2021).

## Highlights

• Methyl-group-modified amino acid analogs (LEU-ME) can localize to lysosomes
• LEU-ME triggers a rapid expansion of the lysosomal compartment
• Fixation procedure is a key step for preserving lysosomal morphology
• Quantification and analysis of lysosomal dimensions

## Graphical abstract

**Figure:**
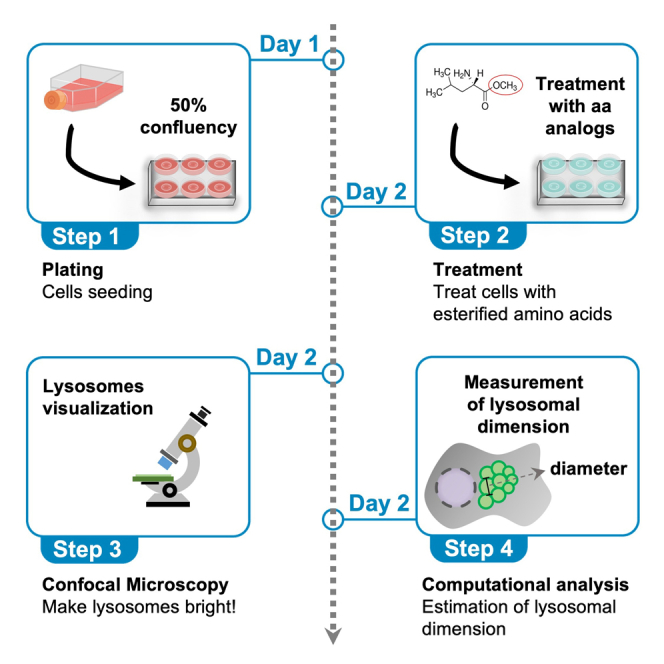

## Before you begin

The protocol below describes the experimental steps required for using HeLa cells stably expressing the lysosomal protein marker Lamp1 tagged with a monomeric form of the green fluorescent protein (mGFP), which allows one to perform the confocal microscopy analysis in living cells. In this regard, the in vivo analysis does represent a key aspect, as it is instrumental in preserving the fragile architecture of the expanded lysosomes. Additionally, here we describe cell fixation methods, which are still able to preserve lysosomal morphology and allow the visualization, as well as the morphometric analysis of the enlarged lysosomes. Importantly, the latter procedure can be deployed even in non-expressing Lamp1-mGFP cell lines, by combining it with a standardized immunofluorescence protocol, which is included into this technical report. Finally, to gain the best and most reproducible effect, it is highly recommended to prepare the amino acid analogs solution at time just before usage.

### Overview

Before starting – covers 1–3 steps: Preparation

Step 1 – covers 1–3 steps: Plate Lamp1-mGFP-expressing HeLa cells on clean, sterile coverslips

Step 2 – covers 4–8 steps: Treat cells with amino acid analogs

Step 3 – covers 9–10 steps: Confocal microscopy acquisition

Step 4 – Quantification and statistical analysis

### Preparation

#### Thaw and culture Lamp1/mGFP-expressing HeLa cell line

**Timing: 1 week**

The aim of this part is to prepare culture cell lines starting from frozen stock aliquots.

1. Thaw cell line from liquid nitrogen or ultra-low temperature fridge.
  a. Thaw cells by putting cryovial in ice and wait 5 min before proceeding.
  b. Transfer cryovial content into a tube containing 5–10 mL of culture medium.
  c. Centrifuge at 1670 rcf for 5 min.
  d. Remove cryopreserving agent-containing supernatant by gentle aspiration.
  e. Gently resuspend cell pellet in an appropriate volume of culture medium and transfer into a cell-culture dish.
  f. Grow cells in a humidified incubator at 37°C and 5% CO_2_.

**CRITICAL:** Do not add any kind of selection agent (i.e.: G-418, Neomycin, or Blasticidin) immediately after thawing.

2. Culture cells until passage 2–3. Split cells every 2–3 days.
  a. Aspirate medium.
  b. Wash cells three times with sterile 1**×** PBS.
  c. Add trypsin and put cells in the incubator for 5 min or up to when cells detach.
  d. Add culture medium and centrifuge cells at 168rcf for 5 min.
  e. Aspirate trypsin-containing medium, resuspend cell pellet in fresh medium and transfer it into a new cell-culture dish.

***Note:*** You can even use non-expressing Lamp1-mGFP cell lines. In such case, you would need to fix cells before staining lysosomes with an anti-Lamp1 antibody (immunofluorescence protocol is described in the step-by-step method details section).

#### Prepare amino acid analog stock solution

**Timing:∼30 min**

3. Prepare 100 mM stock solutions.
  a. Weigh an appropriate amount of powder and transfer it into a 15 mL tube.
  b. Dissolve powder in deionized water by vortexing.
    ***Note:*** these compounds are readily dissolvable in water.
  c. Filter solution with 0.22 μM syringe filters.
  d. Aliquot the stock solution in 1.5 mL tubes and store at −20°C.

**CRITICAL:** Dispose of aliquots after 1–2 freeze/thaw cycles since the biological activity of amino acid analogs is stronger and more reproducible when they are prepared and used freshly.

## Key resources table

**Table:** 

REAGENT or RESOURCE	SOURCE	IDENTIFIER
**Antibodies**		

Mouse monoclonal anti-LAMP1	Sigma-Aldrich	Cat#SAB4700416; RRID:AB_10932380
Mouse Alexa-Fluor (488 and 546) secondary antibodies	Thermo Fisher Scientific-Invitrogen	Cat#A-11029; RRID: AB_138404 Cat#A-11030; RRID: AB_144695

**Chemicals, peptides, and recombinant proteins**		

Bovine serum albumin	Sigma-Aldrich	Cat#A4503
Formaldehyde solution 37%	Sigma-Aldrich	Cat#F15587
Glutaraldehyde solution 70%	Sigma-Aldrich	Cat#G7776
HEPES	Sigma-Aldrich	Cat#H3375
L-Leucine methyl-ester hydrochloride	Sigma-Aldrich	Cat#L1002
L-Alanine methyl-ester hydrochloride	Sigma-Aldrich	Cat#330639
L-Histidine methyl-ester hydrochloride	Sigma-Aldrich	Cat#H15403
Methanol	Sigma-Aldrich	Cat#34860
Saponin	Sigma-Aldrich	Cat#47036

**Experimental models: Cell lines**		

HeLa cells stably expressing Lamp1-mGFP	Kindly provided by prof. Andrea Ballabio (Sbano et al., 2017)	N/A

**Software and algorithms**		

Fiji ImageJ	National Institute of Health	N/A

**Other**		

10 mm-diameter coverslips	VWR	Cat#630-2115
13 mm-diameter coverslips	VWR	Cat#630-2118

## Step-by-step method details

### Step 1: Plate Lamp1-mGFP-expressing HeLa cells on clean, sterile coverslips

**Timing:∼1 h**

Lamp1-mGFP-expressing HeLa cells are seeded on coverslips to allow cell handling in the following steps of the protocol and to allow visualization by confocal microscopy.

1. Trypsinize cells in exponential growth phase, between passage 3 and 20.
  a. Gently remove culture medium.
  b. Wash cells 1 time with pre-warmed PBS (rapid wash).
  c. Add trypsin (or any detaching solution) and put cells in the incubator.
    **CRITICAL:** at the end of trypsinization, make sure cells are at a single-cell level by looking at them under the microscope. Computational analysis becomes difficult if cells form clusters on the coverslip.
  d. Resuspend trypsinized cells in culture medium and remove trypsin by gentle centrifugation and subsequent aspiration of medium.
  e. Resuspend cell pellet in an appropriate volume of culture medium, or at a desired cell concentration.

2. Prepare coverslips.
  a. By using appropriate forceps, put sterile coverslips at the bottom of the well(s).
    **CRITICAL:** We recommend using round 10mm coverslips for 24-multiwell plates or 13mm for 12-multiwell plates, which will minimize any issue (i.e.: breakage, overlay) during their handling. Coverslips must be clean; if they are not, clean them with 1% Sodium Dodecyl Sulfate (SDS), followed by extensive washes with deionized water. Moreover, coverslips must be sterile; if they are not, sterilize them at 200°C for 2h in a heater.
  b. Wash the coverslips 1 time with 1**×** PBS. Make sure they adhere to the bottom of the well(s) by gently pushing them with sterile forceps after cell seeding.

**CRITICAL:** This step is crucial to ensure cells do not grow below the coverslips.

3. Plate cells on coverslips.
  a. Seed cells to have about 50% confluency.
    **CRITICAL:** Do not seed too many cells, if characterized by a high growing rate, as the over-confluency will hamper the consequent computational analysis.
    On the same line, spread cells properly by extensively moving the multi-well (or whichever type of plastic support you are using) in multiple directions, to avoid the formation of cell clusters.
  b. Coverslips can be coated with specific adhesion agents depending on the cell type used.
  c. Using a sterile tip, push gently the coverslips to the bottom of the well since you want cells to adhere on top of the coverslip.
  d. Put cells in the humidified incubator at 37°C and 5% CO_2_ for 16 h before proceeding.

### Step 2: Treat cells with amino acid analogs

**Timing:∼3 h**

Amino acid analogs are methyl ester group-bearing amino acids, which cause the rapid, osmosis-driven, expansion of lysosomes. We tested three types of such compounds on cells (individually): L-Leucine methyl ester hydrochloride, L-Alanine methyl ester hydrochloride, L-Histidine methyl ester hydrochloride. L-Leucine methyl ester hydrochloride caused the strongest effect in our cell models.

4. Treat cells with amino acid analog.
  a. Dilute amino acid analog stock solution in culture medium at the desired concentration.
    ***Note:*** The expansion effect is dose and time-dependent. Use a working concentration between 1mM (mild expansion) and 10mM (maximum expansion) up to at least 2h of incubation to reach the maximum effect. Moreover, if you are using phenol red-containing medium, you will notice a change in color towards yellow. Indeed, amino acid analogs are slightly acidic compounds.
  b. Take cells from point 6d and gently remove culture medium.
  c. Add culture medium supplemented with amino acid analogs and put back cells in the incubator for 2 h.

***Note:*** After having removed culture medium (7b) add the one containing amino acids analogs (7c) without introducing any further cell starvation step.

### Cell fixation

**Timing:∼30 min**

Fixation is the most critical step of the protocol. The membrane of expanded lysosomes is extremely fragile, and their morphology can be strongly affected by the fixation method. Hence, the aim of this section is to describe a detailed protocol based on three different fixation procedures, which allows to preserve at its best the lysosomal morphology.

5. Methanol-based fixation procedure:
  a. Pour pure methanol in a multi-well or in any kind of plastic support you need to use.
  b. Put methanol-containing multi-well at −20°C in a fridge.
  c. Wait approximately for 15 min.
  d. Put multi-well from 6d on ice.
  e. Using the appropriate forceps, transfer coverslips-containing cells into cold methanol.
  f. Wait 5 min.
    **CRITICAL:** Do not extend fixation time over 5 min, as the methanol fixation also promotes membranes permeabilization and, as such, a longer exposure could eventually affect *per se* the lysosomal morphology.
  g. Aspirate methanol and wash with 1**×** PBS by 4 or 5 rapid washes before proceeding with immunostaining step or confocal microscopy analysis.

6. Glutaraldehyde-based fixation procedure:
  a. Aspirate culture medium and wash the cells one time with 1**×** PBS at room temperature (Alcantara-Ortigoza et al., 2010).
  b. Fix the cells by adding a mixture of 4% paraformaldehyde (PFA) and 0.05% glutaraldehyde (GA) prepared in 0.2M HEPES buffer for 10 min (Alcantara-Ortigoza et al., 2010).
  c. Wash the cells once with 4% PFA (prepared in 0.2M HEPES buffer) to remove the residual GA and fix them again by adding 4% PFA and incubate them at RT for 30 min.
  d. Wash the cells three time with 1**×** PBS solution before proceeding with immunostaining step or microscopy analysis.

**CRITICAL:** Upon fixation, cells can be stored at 4°C for few days. If a longer period is needed, it is recommended to seal up the multi-well plate with Parafilm to avoid any evaporation of the PBS solution.

7. Formaldehyde-based fixation procedure:
  a. Aspirate culture medium and wash the cells one time with 1**×** PBS at RT.
  b. Fix the cells by adding formaldehyde solution (3.7% in 1**×** PBS) and let it to act for 30 min at RT.
  c. During incubation, prepare the glycine-based blocking solution (0.1M Gly in 1**×** PBS). This solution must be freshly prepared and cooled in ice before use.
  d. Aspirate fixation solution and replace it with glycine-based blocking solution. Incubate on ice for 5 min.
  e. Wash the cells two times with 1**×** PBS before proceeding with the immunostaining protocol.

### Cell immunostaining of lysosomal endogenous proteins

**Timing:∼3 h**

This section describes the immunostaining method for the endogenous lysosomal marker Lamp1 protein. This is a critical step for both the visualization of enlarged lysosomes by means of confocal microscopy and for the quantitative analysis of their morphological changes (i.e.,: number, size, diameter).

8. In our study, we used anti-Lamp1 antibody to visualize the lysosomal compartment.
  a. After having treated cells with amino acid analogs and proceeded with fixation protocol, cells were permeabilized by using blocking buffer (0.05% saponin, 1% bovine serum albumin in 1**×** PBS) for 15 min at RT.
    ***Note:*** Methanol fixation method already promotes cell permeabilization. Thus, step 6a can be omitted.
  b. During the blocking step, dilute at a 1:500 ratio the anti-Lamp1 antibody in blocking buffer.
  c. Wash cells once with 1× PBS at 25°C.
  d. Mix the diluted antibody by inverting or vortexing, and spin down for a second.
  e. Prepare a rectangle of parafilm on the working bench by spreading a few drops of water between the parafilm and the bench to let it to adhere.
  f. Add 50 μL of the diluted primary antibody solution on the parafilm. This volume is appropriate for 10 mm and 13 mm coverslips. Add more volume for larger coverslips. The right volume is the one that allows the coverslip to float on the drop.
  g. Gently put the coverslip upside-down with the cells in contact with the drops of antibody-containing solution and incubate for 1 h at RT.
    ***Note:*** This drop-based protocol allows one to reduce the consumption of the antibody for each experiment, yet without influencing the efficacy and/or reproducibility of the procedure.
    **CRITICAL:** Before positioning the coverslip on the drops, remove the excess PBS solution that is still adsorbed on the coverslip by letting it touch the surface of a piece of adsorbing paper. This step is fundamental to avoid any further and undesired dilution of the antibody.
  h. Afte the incubation with primary antibody, add 150 μL of 1**×** PBS between parafilm and coverslip to let it float on the drops.
    **CRITICAL:** This step is instrumental for transferring back coverslips from Parafilm to multi-well without damaging or leaving cells on the Parafilm bedding.
  i. Wash cells three times with 1**×** PBS for 5 min each.
  j. Dilute the Alexa Fluor 488-conjugated secondary antibodies at a ratio of 1:500 using blocking buffer.
  k. Mix the diluted secondary antibodies by inverting or vortexing, and spin down for a second.
  l. Add 50 μL drops of the diluted secondary antibodies solution.
    **CRITICAL:** Incubation with secondary antibodies must be performed in the dark. Cover the coverslips with a cover to protect them from light and prevent photobleaching.
  m. Incubate for 45 min at RT.
  n. Wash cells three times with 1**×** PBS 5 min each.
  o. Add 10 μL of mounting solution on the slide.
  p. Dip coverslips in sterile water before putting them on the drop of mounting solution to remove any trace of salts.
  q. Let them dry in a dark chamber before proceeding with microscopy analysis.

### Step 3: Confocal microscopy acquisition

**Timing: 1 day**

In this section we describe in detail the setting and acquisition mode applicable to either living or fixed cells by using the Ultraview VoX spinning disk system (PerkinElmer) (D'Agostino et al., 2018; D'Agostino et al., 2017; D'Agostino et al., 2016) or the LSM700 confocal system (Carl Zeiss) (D'Agostino et al., 2014; D'Agostino et al., 2013), respectively. The protocol described here is applicable for any fluorescent molecule excitable at 488 nm (such as a GFP, FITC, Alexa Fluor 488, etc.), as well as for other fluorescent molecules excitable at any other different wavelengths.

9. Live-imaging acquisition procedure:
  This section of the protocol describes the visualization of the exogenous lysosomal marker Lamp1-mGFP protein. For the live-based visualization, cells must be grown in multi-well with glass-bottom and the microscope must be equipped with a dedicated chamber necessary to maintain temperature and CO_2_ at 37°C and 5%, respectively.
  a. Switch on the microscope and set up parameters to the thermostatic chamber to let it reach the selected temperature and CO_2_.
  b. All samples are observed by using an oil immersion objective (63× 1.4 NA).
  c. The channel is set to 488-nm laser by using the Ultraview VoX spinning disk software.
  d. Adjust the laser power and time exposure. For this study, the variables were adjusted as follows: laser power at 50% and time exposure between 200 and 500 ms.
  e. Researchers should optimize the settings for optimal image intensity.
  f. After capturing, images can be processed using the Ultraview VoX spinning disk software to adjust their intensity.
  g. Capture images from at least 20 different fields for the quantification of lysosomal diameter.
  h. Acquired images are saved as .Tiff, .JPG or .PNG formats and can be visualized for quantitative analysis by using the ImageJ Fiji software.

10. Acquisition procedure from fixed cells:
  This section describes the visualization of stained cells to analyze the endogenous lysosomal marker Lamp1 protein. All images are acquired by confocal microscopy. To use LSM700 software, researchers need to refer to the manufacturer’s manual for the Zeiss LSM700 instrument.
  a. All samples are observed using a confocal microscope equipped with a 63**×** or 40**×** 1.4 NA oil immersion objective. The observation can be made even with a non-confocal microscope.
    **CRITICAL:** Do not use non-oil objectives. The oil is necessary to improve the resolving power of the microscope which is crucial to get high-resolution images that allow the best estimation of the dimension from the smallest to the largest lysosomal structures.
  b. The channel is set to 488-nm laser by using the LSM700 Zeiss software.
  c. Adjust the pinhole, laser power, PMT gain, and offset. For this study, the variables were adjusted as follows: pinhole at 0,42 A.U., laser power at 2.0, PMT gain between 500 and 700, and the offset at −20.
  d. Researchers should optimize the settings for optimal image intensity varying and keeping laser power or PMT gain always under the maximum before bleaching. Anyway, the instructions we have provided on the Lamp1-mGFP expressing cells, the antibody against Lamp1, and the fixation procedures guarantee the optimal outcome which can be easily reached also with other acquisition systems including the non-confocal ones.
  e. After capturing, images can be processed using LSM700 Zeiss software to adjust their intensity.
  f. Capture images from at least 20 different fields of three independent experiments. For a good statistically significant quantification, one need to analyze at least 50 lysosomal diameters for 20 cells/experiment for a total of 3000 lysosomal diameters.
  g. Acquired images are saved as “.lsm5” format and can be opened using LSM700 or ImageJ Fiji software. Images can then be exported to the .Tiff, .JPG, .PNG, and many others for the post-processing analysis.

## Expected outcomes

**Figure 1:**
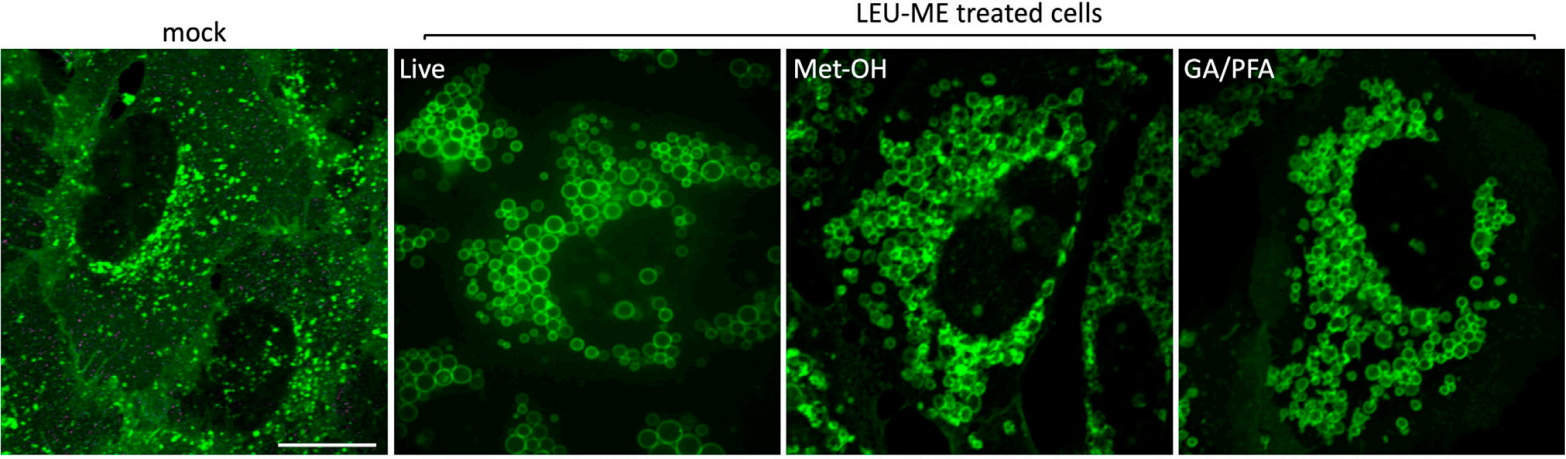
Morphological comparison between enlarged lysosomes observed in living condition or after different fixation procedures HeLa cells, stably expressing the lysosomal marker Lamp1-mGFP, were treated or not (mock cells were treated with only the buffer used for the preparation of the stock solution of the amino acids analogs) for 2 h with amino acid analogs (10 mM LEU-ME) and differently handled (live versus methanol or glutaraldehyde/paraformaldehyde fixation), before being analyzed by confocal microscopy.

The analysis of lysosomal dimension is important to understand how cells respond to the substrate accumulation into the lysosomal compartment. In this regard, this protocol provides a useful method for monitoring lysosomal dimension in either living cells, or upon fixation. Amino acid analogs are characterized by the presence of a methyl ester group, which renders them able to diffuse through any membrane-enclosed compartment, without causing any osmotic effect due to their accumulation. However, the lysosomal compartment possesses a hydrolase that removes the methyl ester group causing the selective accumulation of the amino acid analog within the lysosomal lumen (Reeves, 1979). As such, the aa-analog accumulation causes an osmotic effect, which ultimately triggers lysosome expansion via a membrane fusion-dependent process (Desfougeres et al., 2016; Scerra et al., 2021). Consistent with this, we observed that after 2 h of amino acid analogs accumulation, lysosomes expand appearing like large and perfect spheres (Figure 1). The usage of a transmembrane protein to label the limited membrane of the compartment is instrumental to better define the boundary of a single lysosomal structure and to allow a more accurate measurement of its diameter.

## Quantification and statistical analysis

**Timing:∼3 h**

All images exported in JPEG, Tiff, PNG, and .lsm5 or .lsm7 format can be directly opened in Fiji ImageJ software for the consequent quantitative analysis.

**Figure 2:**
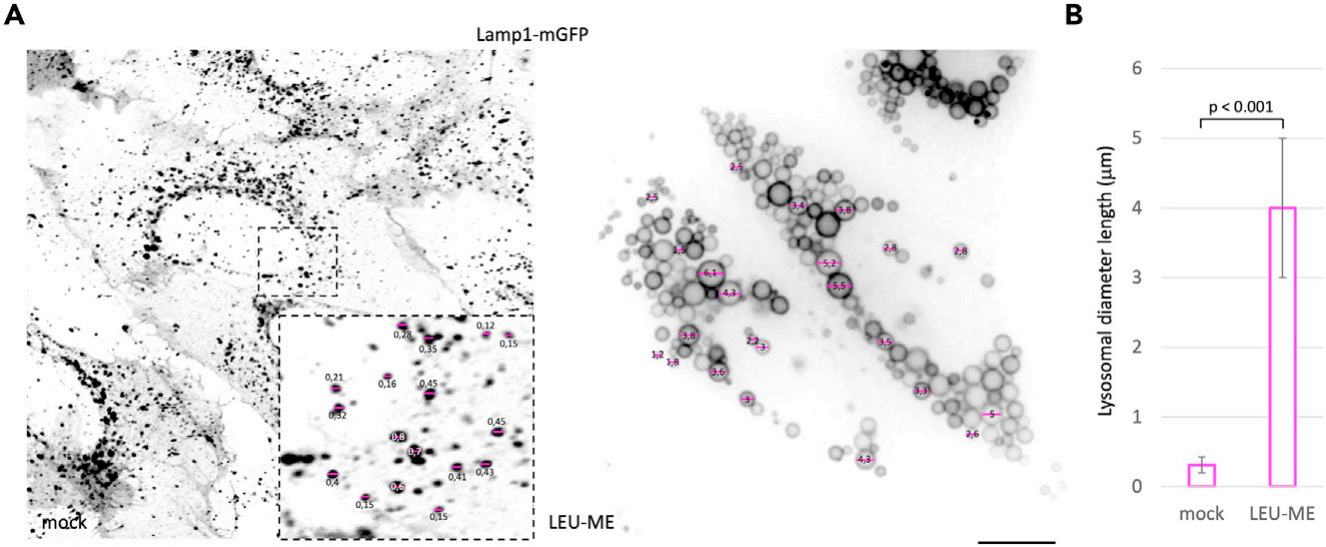
Measurement of lysosomal diameter Living HeLa cells stably expressing Lamp1-mGFP were grown in 12-multiwell plate with glass-bottom, treated or not with LEU-ME 10 mM for 2 h, and imaged by using a Ultraview VoX spinning disk microscope. Some representative lysosomal diameters are indicated by magenta lines and their lengths have been estimated by ImageJ Fiji software and shown in μm scale (A). The histogram shows the average length of lysosomal diameters from 3000 measurements from 20 cells for each of the three independent experiments (B). Statistical analysis was performed by One-way ANOVA. Scale bar: 20 μm.
4. Go to “analyze” and click on “measure”.
5. A table in a new window will appear. In the table are reported different measurements referring to the lines dawn on the picture including area, mean fluorescence intensity, and length (this number corresponds to the number of pixels contained in the drawn line).
6. For the statistical significance, we measure the diameter of 50 lysosomes from 20 cells for each of the three experiments performed for a total of 3000 lysosomal diameters.
7. Before proceeding with the estimation of the real length expressed in μm, repeat the same measurement on the scale bar (for this protocol a 20 μm scale bar was used).
8. To convert the length of each diameter, expressed in number of pixels, into μm scale, the following formula was used: (n pixels diameter/n pixels scale bar) **×** 20 μm.

***Note:*** Since the smaller lysosomal size of the mock-treated cells, we suggest performing the measurement of the lysosomal diameters on the zoomed images already opened in Fiji software. Indeed, zoomed images conserve the pixel dimension of the original images and it allows better visualization of the florescent signal extension necessary to correctly draw the diameter line.

1. Open the image file in Fiji program.
2. Click on “straight” in the tools bar.
3. Draw a line corresponding to the diameter of a selected enlarged lysosome by using the Lamp1 fluorescent signal as a reference of the limiting membranes of each selected structure (Figure 2A).

9. Once converted, mean values were plotted on the histogram (Figure 2B). Standard deviations were determined, and significance assessed by using One-way ANOVA .

## Limitations

Amino acid analogs accumulate within lysosomes because of intra-luminal cleavage of methyl ester groups which render them membrane permeable, thereby limiting their export from the lysosomes back to the cytoplasm by free diffusion. In this protocol, we analyzed lysosomal size after 2 h of treatment with LEU-ME. This timing was sufficient to promote the lysosomal expansion whose enlarged size was stable up to 3 h. However, from this time point forward, lysosomes start to rescue their initial size. Indeed, once accumulated, cleaved amino acid analogs can be exported by lysosomal transporters into the cytosol, thereby dissipating the osmotic effect and the enlarged size of the organelle. Thus, this protocol is applicable only for a short time treatment whereas, for a longer time, a microfluidic chamber needs to be employed to constantly replenish the extracellular solution and keep constant the high concentration of the amino acid analogs into the lumen of lysosomes.

## Troubleshooting

### Problem 1

**Figure 3:**
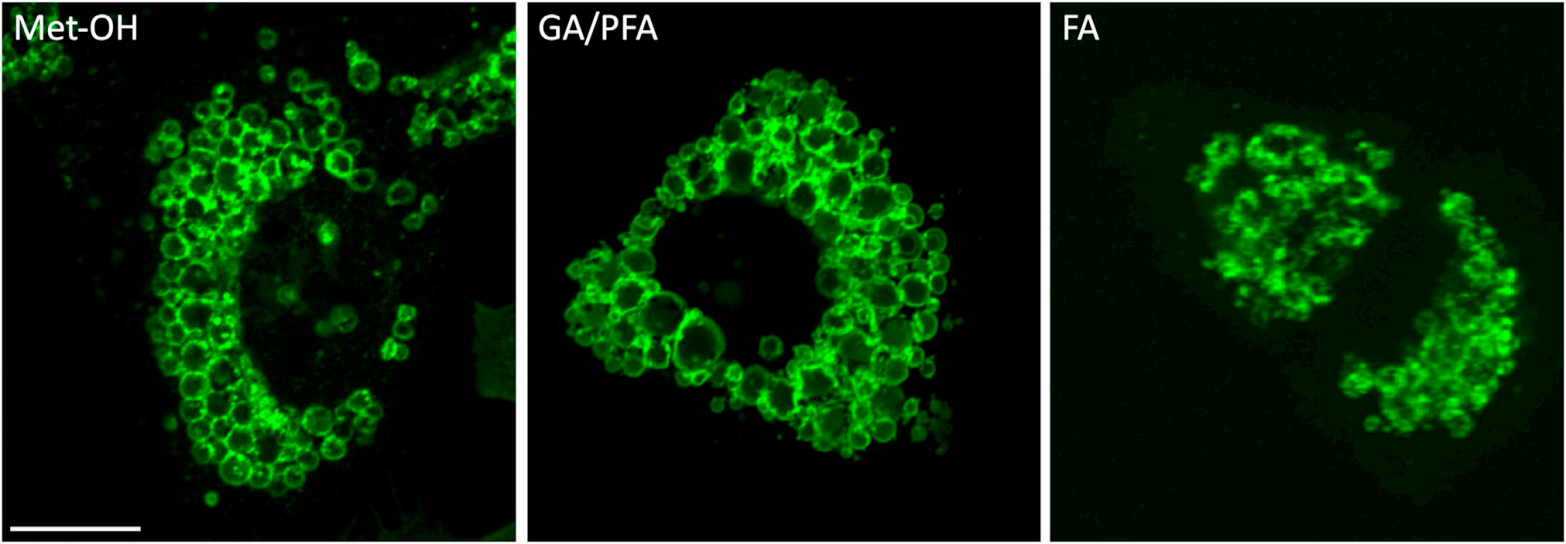
Comparison between good versus bad outcome of the fixation procedure on the lysosomal morphology HeLa cells were grown on coverslips and fixed with different chemical reagents indicated in each panel and lysosomes were labeled by using a specific antibody against the lysosomal protein marker Lamp1. Met-OH: methanol; PFA: para-formaldehyde; FA: formaldehyde. Scale bar: 20 μm.

The fixation procedure affects the morphology of the enlarged lysosomes (Figure 3. Please compare the FA panel on the right respect to the Met-OH and GA/PFA panels) (referred to the sections from 5 to 7).

### Potential solution

Enlarged lysosomes are highly sensitive to the fixation procedure and formaldehyde solution (3,7% formaldehyde dissolved in 1**×** PBS) determines a severe alteration of the lysosomal morphology (Figure 3, right panel). Such a morphological alteration is evident either in Lamp1-mGFP stably-expressing cells or in cells expressing the endogenous levels of Lamp1 protein and stained by using a specific antibody. We therefore strongly recommend avoiding using formaldehyde and to opt (also in consideration of the eventual combination of antibodies to be used along with the anti-Lamp1) for one of two alternative procedures we have established, which have proved successful in preserving at its best the lysosomal morphology.

### Problem 2

Problem to take coverslips from wells (referred to the section 8 g).

### Potential solution

We recommend using a 10 mm or 13 mm coverslip for 12 well plates. 10 mm coverslip can be used also for 24 but not 48 well plates. As a rule, we suggest using coverslips with an area about half that of one of the corresponding wells.

### Problem 3

Before putting coverslips on the antibody-containing drops, by distraction, one could forget on which side of the coverslip the cells are (referred to the section 8 g).

### Potential solution

Keeping the coverslip with forceps, gently scrape one time a side of the coverslip by using a tip. The appearance of a trace indicates the side of the coverslip where the cells were seeded.

### Problem 4

After the incubation with the primary antibody, the drop was dry, and the coverslip remains attached to the parafilm (referred to the section 8 h).

### Potential solution

This problem should not arise if the lab is equipped with an automatic system that guarantees constant control of the temperature over the entire year. However, many labs do not possess these systems and the room temperature could vary depending on the seasons. For this reason, during summer, we suggest improving the volume of the drops used. Alternatively, we suggest building a humid chamber that prevents drops to dry and allows even longer incubation times. Moreover, we suggest adding 100–150 μL of PBS 1**×** to let the coverslip float on the drop. This will render the handling of the coverslip much easier (the last suggestion is shown in the figure).

### Problem 5

Once the mounting medium was dry, many bubbles appear, and the microscopy analysis is largely impaired (referred to the section 8 q).

### Potential solution

Before letting the mounting medium becomes dry, we recommend pushing with a tip on the coverslips while aspirating the mounting excess. In this way, all the bubbles should be removed.

## Resource availability

### Lead contact

Further information and requests for resources and reagents should be directed to and will be fulfilled by the lead contact, Massimo D’Agostino (massimo.dagostino@unina.it).

### Materials availability

This study did not generate new unique reagents.

## Data Availability

This study did not generate datasets or code.
